# Supplementary material for: Environmental sex determination in the cyst nematode Globodera pallida defaults to male development
Source: BMC Biol. 2026 Jul 3;24:204. doi: 10.1186/s12915-026-02671-y (PMC13632290; doi:10.1186/s12915-026-02671-y)
Supplement: Supplementary file 1 [file 12915_2026_2671_MOESM2_ESM.pdf]

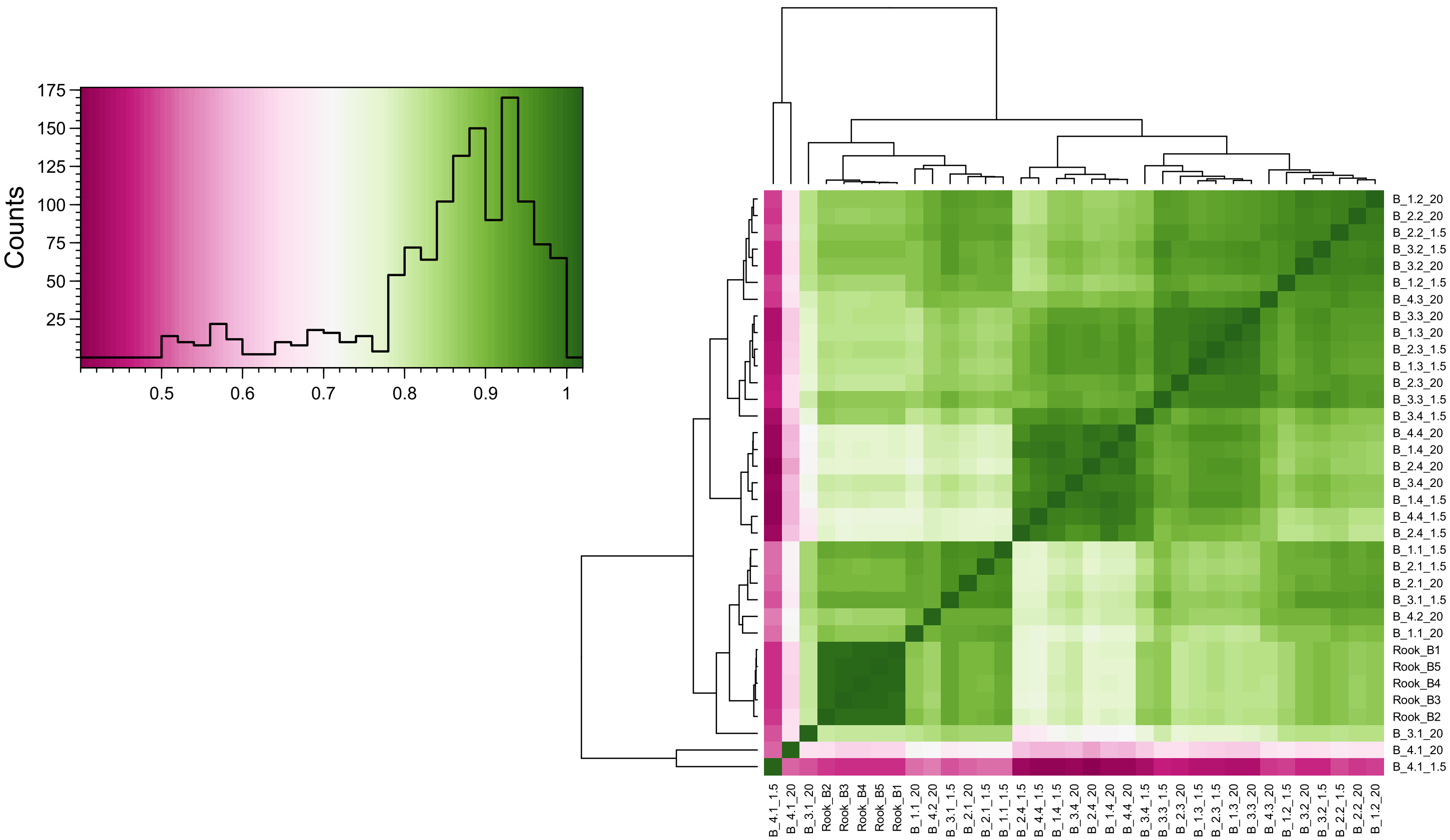
Counts
175
150
125
100
75
50
25
0.5
0.6
0.7
0.8
0.9
1
B_1.2_20
B_2.2_20
B_2.2_1.5
B_3.2_1.5
B_3.2_20
B_1.2_1.5
B_4.3_20
B_3.3_20
B_1.3_20
B_2.3_1.5
B_1.3_1.5
B_2.3_20
B_3.3_1.5
B_3.4_1.5
B_4.4_20
B_1.4_20
B_2.4_20
B_3.4_20
B_1.4_1.5
B_4.4_1.5
B_2.4_1.5
B_1.1_1.5
B_2.1_1.5
B_2.1_20
B_3.1_1.5
B_4.2_20
B_1.1_20
Rook_B1
Rook_B5
Rook_B4
Rook_B3
Rook_B2
B_3.1_20
B_4.1_20
B_4.1_1.5
B_4.1_1.5
B_4.1_20
B_3.1_20
Rook_B2
Rook_B3
Rook_B4
Rook_B5
Rook_B1
B_1.1_20
B_4.2_20
B_3.1_1.5
B_2.1_20
B_2.1_1.5
B_1.1_1.5
B_2.4_1.5
B_4.4_1.5
B_1.4_1.5
B_3.4_20
B_2.4_20
B_1.4_20
B_4.4_20
B_3.4_1.5
B_3.3_1.5
B_2.3_20
B_1.3_1.5
B_2.3_1.5
B_1.3_20
B_3.3_20
B_4.3_20
B_1.2_1.5
B_3.2_20
B_3.2_1.5
B_2.2_1.5
B_2.2_20
B_1.2_20
